# New constitutive model and hot processing map for A100 steel based on high-temperature flow behavior

**DOI:** 10.1016/j.heliyon.2024.e40823

**Published:** 2024-12-02

**Authors:** Chaoyuan Sun, Yi Qin, Yang Liu, Guiqian Xiao, Jie Zhou, Jiansheng Zhang

**Affiliations:** a Chongqing Key Laboratory of Advanced Mold Intelligent Manufacturing, College of Materials Science and Engineering, Chongqing University, Chongqing, 400044, China; b China National Erzhong Group Deyang Wanhang Die Forging Co Ltd, Deyang, 618013, China; c Chongqing Jiepin Technology Co. Ltd., Chongqing, 400000, China

**Keywords:** Constitutive model, A100 steel, Dynamic recrystallization, Hot processing map

## Abstract

To predict the flow behavior and identify the optimal hot processing window for A100 steel, a constitutive model and a hot processing map were established using true stress-strain data extracted from isothermal compression tests performed at temperatures ranging from 1073 to 1353 K and strain rates varying between 0.01 and 10 s^−1^. The results indicate a strong linear trend between the logarithmic stress and the reciprocal of temperature, along with a significant quadratic relationship between the logarithmic stress and logarithmic strain rate. With a correlation coefficient of 0.9913, the new constitutive model demonstrates an excellent predictive capability for the flow behavior of A100 steel. A significant dynamic recrystallization occurs when the energy dissipation rate exceeds 25 %, resulting in a uniform equiaxed grain structure after deformation. The unstable regions primarily occur in high strain rate and low-temperature zones, where the microstructures are typically coarse and elongated. This structural characteristic adversely affects the mechanical properties. Avoiding these conditions during hot forming of A100 steel is crucial. The optimal hot forming windows for A100 steel are achieved within a temperature range of 1153–1353K and a strain rate range of 0.01–10 s^−1^, where complete dynamic recrystallization occurs.

## Highlights

• True stress-strain curves of A100 steel were obtained through isothermal compression tests.
• The stress of A100 steel is most significantly influenced by strain rate, temperature, and strain.
• The new constitutive model outperforms traditional models in predicting flow behavior.
• The hot processing map and microstructural analysis are used to establish conditions for equiaxed grain formation.
• Hot processing window is identified within a temperature range of 1153–1353K and a strain rate of 0.01 to 10s-1.

## 1 Introduction

A100 steel, alloyed with C, Cr, and Mo elements, is widely used in various components of aircraft, including landing gear, arresting hooks, catapult launch bars, and turbine shafts [1,2]. Typically, these parts are formed through hot forging. Consequently, studying the high-temperature flow behavior of A100 steel is essential for enhancing the forming process and developing accurate numerical models for simulation purposes. In the past, various empirical, semi-empirical, phenomenological, and physics-based constitutive models have been developed to characterize the high-temperature flow behavior. Phenomenological models, including the Arrhenius(AH) model [[3], [4], [5]], Johnson-Cook(JC) model [6], and Zerilli-Armstrong(ZA) model [7,8], are widely applied in commercially FE software to define the constitutive relationship. Their popularity stems from their simplicity, as they require fewer material constants and minimal experimental data [9]. Some studies have focused on Aermet100 steel. For example, Ji et al. [10] created an artificial neural network (ANN) model to forecast the high-temperature rheological behavior of Aermet100 steel. The ANN model showed effective and precise predictions of the flow stress, utilizing input parameters like strain rate, strain, and temperature. This model demonstrated strong extrapolation capabilities within the vicinity of the training domain and showed excellent tolerance to noise. Yuan et al. [11] conducted hot compression tests on AerMet100 steel and developed a constitutive equation using double-multivariate nonlinear regression to analyze the true stress-strain data and the microstructural changes. Hot processing maps, derived from the Malas stability criterion, were used to identify the optimal deformation conditions. Observations indicated the presence of shear bands in the unstable region and dynamic recovery in the stable region, with deformation instability being attributed to the formation of adiabatic shear bands driven by the combined effects of temperature, strain rate, and deformation. Qiao et al. [12] developed a constitutive model and a hot working map for Aermet100 steel, based on the experimental true stress-strain data. The maps also identified instability zones in flow behavior, enabling the identification of distinct hot deformation characteristics in different zones of Aermet100 steel. Yuan et al. [13] formulated constitutive equations for high-temperature conditions using both the initial Johnson-Cook (JC) model and a modified version of the JC model. Their results indicated that, in terms of predictability and the number of material constants, the modified JC model proved to be an excellent option for predicting the flow behavior of Aermet100 steel within the studied temperature range. Zhao et al. [14] proposed dynamic recrystallization (DRX) models, incorporating volume fraction and grain size, through a series of isothermal hot compression tests aimed at accurately predicting microstructural evolution. The excellent correlation between the predicted and experimental results confirms the reliability of the developed DRX models, suggesting their potential for quantitatively forecasting the microstructure changes of AerMet100 steel during thermo-mechanical processing.

Phenomenological models are recognized for their simplicity, with few parameters and ease of application. However, research on the constitutive equations and hot processing maps for A100 steel remains scarce. To predict the flow behavior and identify the optimal hot processing parameters for A100 steel, a new constitutive equation and hot processing map were developed based on flow stress data from compression experiments conducted at temperatures between from 1073 to 1353 K and strain rates from 0.01 to 10 s⁻^1^.

## 2 Materials and methods

### 2.1 Material

**Fig. 1:**
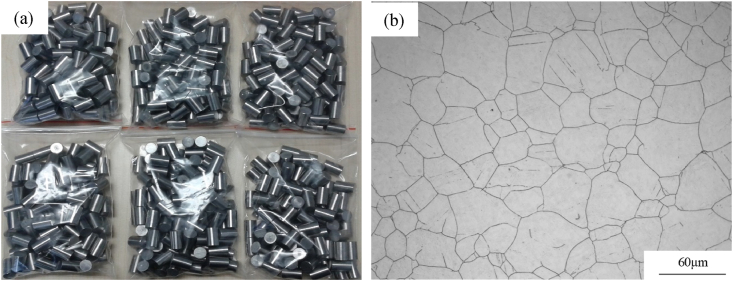
A100 steel experimental specimen: (a) cylindrical test specimen, (b) initial microstructure.

**Fig. 2:**
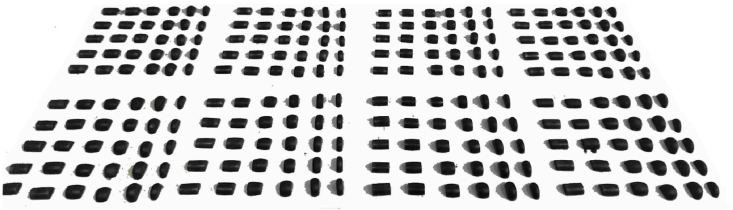
A100 steel specimen after isothermal compression.

A100 steel is an ultrahigh-strength alloy known for its secondary hardening characteristics, featuring a high concentration of cobalt (Co), nickel (Ni), molybdenum (Mo), and chromium (Cr). The detailed elemental composition of A100 steel is listed in Table 1. This steel provides notable benefits, including exceptional strength, superior toughness, and outstanding corrosion resistance. Fig. 1(a) shows the cylindrical test specimen, while Fig. 1(b) presents the initial microstructure. The initial average gain size is approximately 50 μm, with an equiaxed grain structure. Fig. 2 shows the compressed specimen.

**Table 1:** Weight percent chemical composition of A100 steel (%).

C	Mn	Ni	Co	Cr	Mo	Si	Fe
0.24	≤0.10	11.50	13.4	3.22	1.25	0.11	Bal.

### 2.2 Experiment methods

**Fig. 3:**
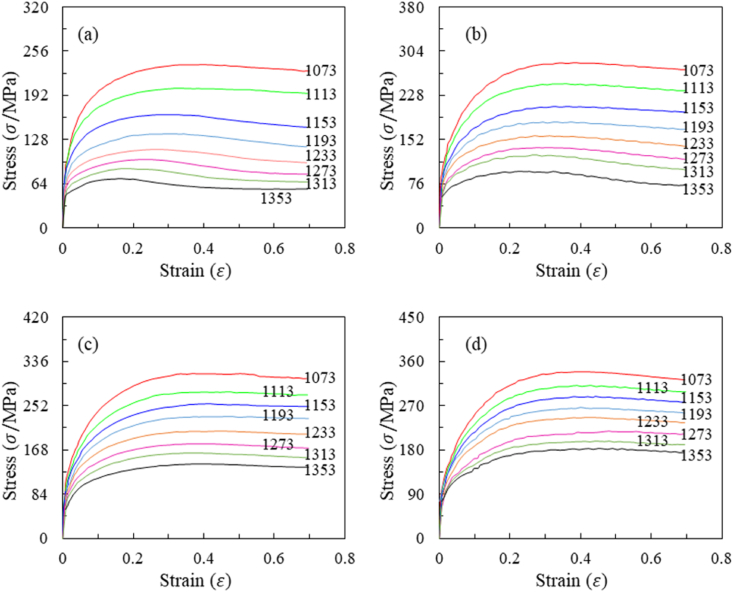
True stress strain curves of A100 steel in hot compression at strain rates of (a) 0.01 s^−1^, (b) 0.1 s^−1^, (c) 1 s^−1^, (d) 10 s^−1^.

Isothermal, constant strain rate compression tests were performed using a Gleeble-3500. The compression specimens, measuring 10 mm in diameter and 15 mm in length, were fabricated with flat-bottomed grooves on their end faces to hold graphite lubricant, thereby minimizing the interface friction between the tools and the metal. The compression experiments were conducted within a temperature range of 1073–1353 K (with a 40 K interval), a strain rate range of 0.01–10 s⁻^1^ (with a 10-fold interval), and a true strain range of 0–0.69. Throughout these experiments, stress-strain data at varying temperatures and strain rates were automatically collected, as depicted in Fig. 3. The compression specimens were first heated to the deformation temperature at a rate of 10 K/s and then held at this temperature for 5 min to ensure thermal equilibrium. Subsequently, the isothermal compression tests were performed. Once deformation was complete, the specimens were rapidly water-quenched to room temperature to preserve the deformed microstructure. To examine the post-deformation microstructure, the water-quenched specimens were sectioned longitudinally (as shown in Fig. 2), and the cross-sections were prepared for metallographic analysis. This process included grinding with 200#, 400#, 600#, 800#, 1000#, 1200#, and 1400# sandpapers for coarse grinding, followed by fine grinding, and then polishing using a polishing machine. Finally, the specimens were etched using a suitable etchant, and their microstructures were analyzed using an optical microscope.

As demonstrated in Fig. 3(a), (b), (c), and **(d)**, A100 steel exhibits a distinct stress peak within the temperature range of 1073–1353 K and across strain rates from 0.01 to 10 s⁻^1^. This indicates that dynamic recovery or recrystallization softening takes place under these deformation conditions. As the strain rate decreases, the spread in the stress-strain data increases, reflecting a greater influence of temperature on the softening behavior of A100 steel. This is because lower strain rates provide more time for dynamic recrystallization to occur, allowing temperature to play a dominant role in the process. In contrast, at higher strain rates, the limited time for recrystallization reduces the effect of temperature fluctuations. Therefore, the stress response of A100 steel is governed by the combined effects of temperature, strain rate, and strain within the specified testing parameters.

To facilitate the subsequent investigation of how strain rate, deformation temperature, and true strain affect the material's true stress, the experimental data were interpolated and discretized. The true stress-strain data at various temperatures and strain rates were segmented and interpolated into 10 equal intervals within the strain range of 0.04–0.69. The resulting data are shown in Table 2.

**Table 2:** Stress data at different temperature, strain, and strain rate.

Strain (ε)	Strain Rate ($\dot{\varepsilon }$/s^−1^)	Temperature (T/K)							
		1073	1113	1153	1193	1233	1273	1313	1353
0.04	0.01	153.00	140.50	118.54	101.52	87.36	76.35	67.23	56.89
	0.1	165.26	150.14	130.88	122.18	110.01	93.11	88.47	72.21
	1	173.84	160.22	148.01	138.43	126.24	113.79	104.07	90.68
	10	175.55	166.96	158.52	151.78	140.76	126.56	120.12	113.14
0.11	0.01	201.87	177.43	148.04	122.07	104.09	90.98	80.91	69.22
	0.1	231.65	207.38	175.69	160.42	140.14	120.58	110.17	88.45
	1	249.85	223.00	205.22	187.75	167.86	148.85	134.93	117.65
	10	262.74	241.82	225.65	210.81	195.43	168.63	155.37	143.99
0.18	0.01	221.72	192.03	158.84	133.30	111.35	97.45	85.52	70.19
	0.1	263.45	231.91	195.78	175.07	150.91	133.61	119.72	95.41
	1	283.58	252.24	231.07	210.46	186.54	165.88	150.09	129.57
	10	305.97	280.60	261.13	242.85	224.66	193.80	179.91	166.12
0.26	0.01	231.44	199.16	163.11	136.49	114.33	98.36	83.91	65.79
	0.1	277.13	242.74	204.99	180.87	156.79	138.16	124.07	96.49
	1	302.84	270.19	245.16	223.10	197.34	174.63	158.23	136.72
	10	327.44	299.08	277.71	256.81	238.22	208.34	190.01	175.65
0.33	0.01	236.06	201.95	163.40	136.56	112.52	94.57	78.96	61.34
	0.1	282.27	246.75	208.66	182.44	157.48	138.49	123.21	96.07
	1	311.98	276.01	252.30	229.27	202.04	178.57	161.45	140.76
	10	337.30	307.83	285.66	262.99	243.64	212.27	194.11	179.55
0.40	0.01	236.50	201.25	160.90	134.28	109.22	89.10	73.41	58.30
	0.1	282.70	246.38	208.08	181.77	156.17	136.28	118.72	91.08
	1	312.53	277.37	256.07	230.96	202.94	179.56	161.86	141.70
	10	339.45	309.52	287.78	267.44	245.96	215.98	196.18	180.45
0.47	0.01	234.35	200.29	156.11	130.17	104.38	83.82	70.20	56.87
	0.1	281.19	244.38	206.69	178.93	153.41	132.58	113.52	85.41
	1	311.59	277.85	254.33	231.13	203.21	178.30	160.25	140.74
	10	337.62	308.99	287.49	265.44	245.21	218.48	196.59	180.96
0.55	0.01	232.04	199.14	152.17	125.79	99.81	80.40	68.32	55.94
	0.1	278.49	241.45	204.03	176.29	149.41	127.39	107.97	79.69
	1	310.57	275.86	252.82	230.16	201.37	176.07	158.51	138.94
	10	333.51	305.67	284.86	262.71	243.01	217.55	194.95	180.15
0.62	0.01	229.97	196.62	148.55	121.76	97.18	78.14	67.10	55.94
	0.1	274.77	238.74	201.47	173.05	145.42	122.73	103.72	75.40
	1	305.80	273.96	252.07	228.46	199.24	173.30	155.66	136.99
	10	328.50	302.11	280.90	259.08	239.86	214.73	192.10	177.12
0.69	0.01	227.08	194.71	145.55	118.09	95.08	77.56	66.36	56.12
	0.1	271.82	235.60	199.26	169.95	141.07	119.18	100.02	73.38
	1	303.53	272.30	251.40	227.43	197.91	171.82	153.65	135.78
	10	323.51	298.24	277.90	256.36	236.04	212.99	190.12	174.22

## 3 Results and discussion

### 3.1 Constitutive models

#### 3.1.1 New equation

Based on the discrete data presented in Table 2, a three-factor analysis of variance (ANOVA) was performed to assess the significance of the influence of each factor on stress [15]. The analysis results, presented in Table 3, reveal the relative impact of individual factors such as strain rate, temperature, and strain, along their interactions. The significance of these factors and their interactions not only highlights their individual contributions but also offers insights into how these interactions modify the overall stress behavior of A100 steel. According to Table 3, the significance order of the factor's affecting stress is as follows: strain rate, temperature, strain, interaction between strain and strain rate, interaction between strain and temperature, and interaction between strain rate and temperature. The F-values clearly indicate that all factors significantly affect stress. Notably, strain rate and temperature emerge as the most influential factors, underscoring their critical roles in determining stress behavior. This suggests that controlling these factors is essential for optimizing material performance under various conditions. The interaction effects also provide valuable insights into how combined factors influence stress, which is crucial for understanding material behavior in real-world applications.

**Table 3:** ANOVA Analysis table.

Source Item	Sum Square	df	Mean Square	F	Prob > F
Strain	149215.90	9	16579.50	2568.86	1.85E-192
Strain Rate	515942.70	3	171980.90	26646.95	5.73E-248
Temperature	847162.10	7	121023.20	18751.49	6.89E-265
Strain∗Strain Rate	31387.30	27	1162.50	180.12	5.93E-120
Strain∗Temperature	26595.30	63	422.10	65.41	1.46E-100
Strain Rate ∗Temperature	8587.40	21	408.90	63.36	7.01E-74
Error	1219.80	189	6.50		
Total	1580111.00	319			

The experimental curve in Fig. 3 illustrates the lower significance of strain compared to strain rate and temperature. After reaching the peak strain, the curve exhibits a horizontal and slightly decreasing trend, indicating that the influence of strain on stress diminishes once strain reaches a certain value.

**Fig. 4:**
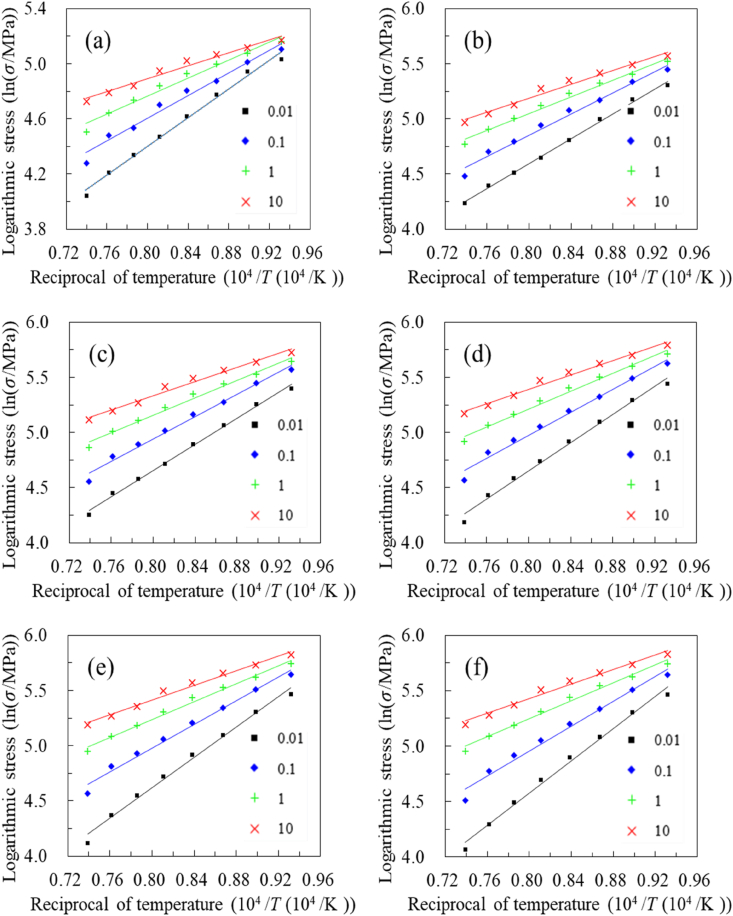

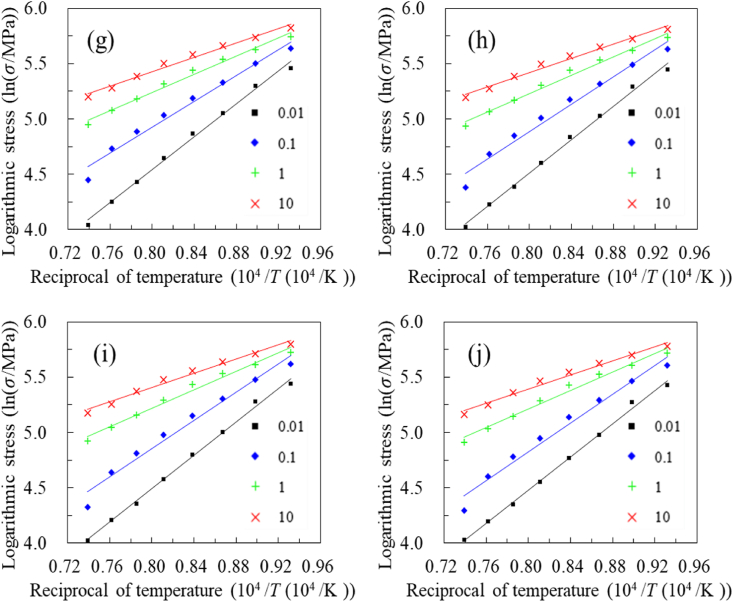
Linear fitting of logarithmic stress and reciprocal temperature at varying strains: (a) strain 0.04, (b) strain 0.11, (c) strain 0.18, (d) strain 0.26, (e) strain 0.33, (f) strain 0.40, (g) strain 0.47, (h) strain 0.55, (i) strain 0.62, (j) strain 0.69.

To investigate the constitutive relationship of A100 steel, the logarithmic stress and reciprocal temperature corresponding to the same strain at different strain rates are regressed. According to Arrhenius model and Hensel-Spittel model, There are linear relationships between the logarithmic stress, logarithmic strain, and reciprocal temperature [[3], [4], [5]]. To reduce material parameters and enhance the extrapolation performance, the study begins with a linear relationship, focusing on the correlation between the logarithmic stress and the reciprocal of temperature. As shown in Fig. 4, a strong linear correlation exists between the logarithmic stress and reciprocal temperature across all strain and strain rate levels. The lowest correlation coefficient, which is greater than 0.98, is observed at a strain rate of 10 s⁻^1^.

As shown in Fig. 4 (a)–**(j)**, several patterns can be identified. Firstly, a strong linear relationship is observed between logarithmic stress and reciprocal temperature across various strains and strain rates. Secondly, at a given strain, a higher strain rate is associated with a smaller slope. Finally, with constant strain, the coverage ranges of trend lines for all strain rates gradually diminish as temperature decreases. These indicate that as the temperature decreases, the effect of strain rate on stress becomes less significant, and conversely, as the temperature increases, the influence of strain rate becomes more pronounced. Based on these observations, the relationship between the logarithmic stress and the reciprocal of temperature can be approximated by Eq. (1).

(1) $$\ln\;\sigma =m_{0}+\frac{m_{1}}{T}$$

where $m_{0}$ and $m_{1}$ represent material parameters.

**Fig. 5:**
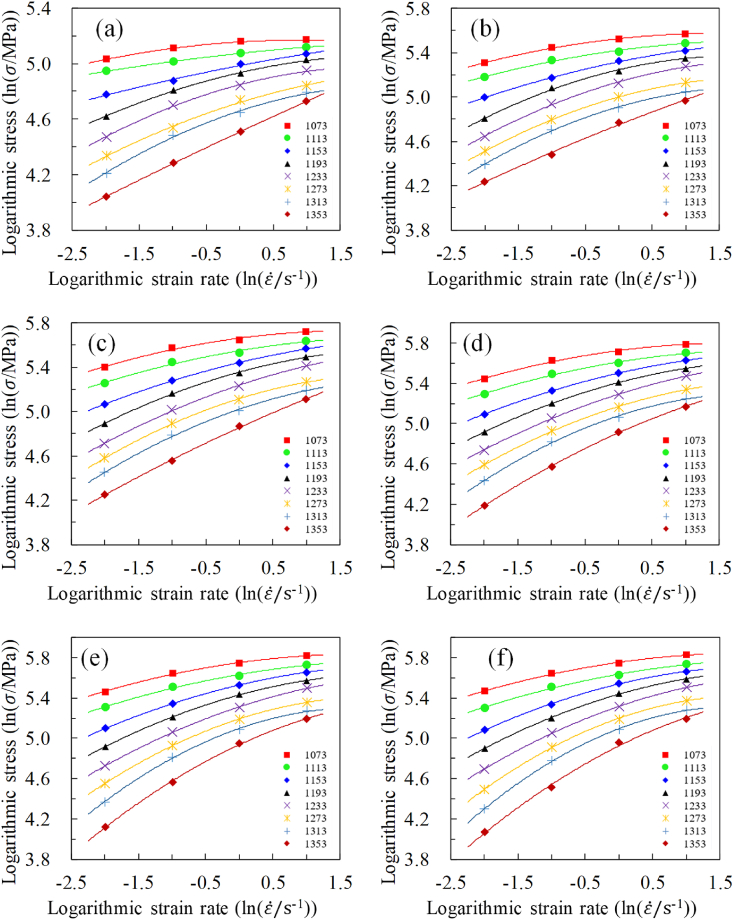

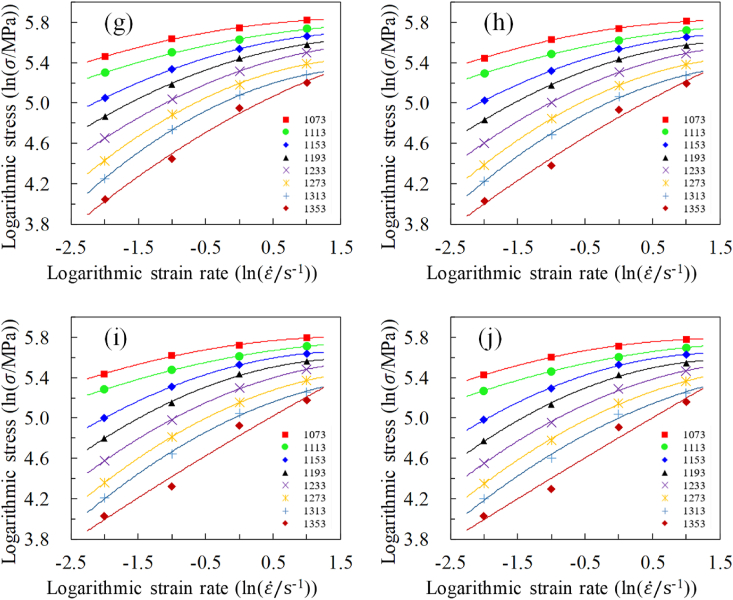
The quadratic fitting between logarithmic stress and strain rate at varying strains: (a) strain 0.04, (b) strain 0.11, (c) strain 0.18, (d) strain 0.26, (e) strain 0.33, (f) strain 0.40, (g) strain 0.47, (h) strain 0.55, (i) strain 0.62, (j) strain 0.69.

Similarly, additional analysis is needed to explore the correlation between strain rates and stress. The logarithmic stress and logarithmic strain rate data for the same strain but at varying strain rates were plotted on a single graph, as shown in Fig. 5 (a) to (j). This approach allows for a detailed examination of how stress responds to changes in strain rate. The observed weak linear relationship suggests that while a simple linear model may not fully capture the stress-strain rate relationship, a quadratic model provides a better approximation. This refinement enhances the accuracy of our material model and highlights the complex nature of the stress-strain rate relationship. It can be observed that a weak linear correlation exists between logarithmic stress and logarithmic strain rate. On one hand, relying solely on linear relationships may lead to insufficient predictive accuracy. On the other hand, simplifying the material model as much as possible necessitates avoiding excessive addition of material parameters. Hence, a quadratic relationship was assumed between logarithmic stress and logarithmic strain rate. In Fig. 5(a)–(j), the solid line represents the outcome of a quadratic polynomial approximation, which shows a high accuracy in the quadratic approximation, with the correlation coefficient remaining above 0.98.

Firstly, a strong quadratic relationship exists across various strains and strain rates. Secondly, at the same strain, a higher temperature corresponds to a larger derivative at the same strain rate level. Finally, when the strain remains constant, the range of trend lines corresponding to all strain rates gradually diminishes with increasing strain rate. This suggests that with an increase in strain rate, the effect of temperature on stress decreases, and vice versa. Based on these observed patterns and classical models (such as the Arrhenius model and Hensel-Spittel models), Eq. (2) can be used to approximate the relationship between logarithmic stress and logarithmic strain rate.

(2) $$\ln\;\sigma =n_{0}+n_{1}\;\ln\dot{\varepsilon }+n_{2}\;\ln^{2}\dot{\varepsilon }$$

where $n_{0}$, $n_{1}$ and $n_{2}$ represent material parameters.

Based on the preceding analysis, the relationship between logarithmic stress and the reciprocal of temperature is assumed to be linear, whereas the relationship between logarithmic stress and logarithmic strain rate is assumed to follow a quadratic form. In fact, a higher-order model results in greater accuracy. However, to maintain extrapolation performance and avoid oscillatory phenomena, a model with as low an order as possible should be selected. Moreover, the interaction between temperature and strain rate has a minimal effect on stress, particularly when compared to the individual impacts of temperature and strain rate (as indicated by the F-values in Table 3). Therefore, the fundamental equation among stress, temperature, and strain rate can be expressed as Eq. (3).

(3) $$\ln\;\sigma =f\left(\frac{1}{T}\right)f\left(\ln\dot{\varepsilon }\right)=\left(m_{0}+\frac{m_{1}}{T}\right)\left[n_{0}+n_{1}\;\ln\dot{\varepsilon }+n_{2}\;\ln^{2}\dot{\varepsilon }\right]$$

The expanded form of **Eq.** (3) is given by Eq. (4).

(4) $$\ln\;\sigma =m_{0}n_{0}+m_{0}n_{1}\;\ln\dot{\varepsilon }+m_{0}n_{2}\;\ln^{2}\dot{\varepsilon }+{n_{0}m}_{1}\frac{1}{T}+{n_{1}m}_{1}\frac{1}{T}\ln\dot{\varepsilon }+m_{1}n_{2}\frac{1}{T}\ln^{2}\dot{\varepsilon }$$

After substituting the parameters, equation (4) becomes is transformed into Eq. (5).

(5) $$\ln\;\sigma =k_{0}+k_{1}\;\ln\dot{\varepsilon }+k_{2}\frac{1}{T}+k_{3}\frac{1}{T}\ln\dot{\varepsilon }+k_{4}\;\ln^{2}\dot{\varepsilon }+k_{5}\frac{1}{T}\ln^{2}\dot{\varepsilon }$$

where $k_{0}-k_{5}$ are material parameters related to strain.

Using the discrete data from Table 2 and applying it to Eq. (5) for multiple linear regression, the material parameters for different strains were determined, as presented in Table 4.

**Table 4:** Material parameters at different strains.

	Material parameters						
Strain	$k_{0}$	$k_{1}$	$k_{2}$	$k_{3}$	$k_{4}$	$k_{5}$	R-squared
0.04	2.2795	0.3345	3111.7336	−344.4006	−0.0274	29.2232	0.9860
0.11	2.0529	0.3067	3752.6704	−298.5201	−0.0279	26.0502	0.9912
0.18	2.0183	0.3548	3937.9092	−344.3849	−0.0226	20.0652	0.9924
0.26	2.0247	0.3955	3997.2113	−386.7361	−0.0266	24.0852	0.9916
0.33	2.0103	0.4089	4043.0826	−400.1191	−0.0420	41.0975	0.9916
0.40	2.0079	0.4626	4053.3193	−459.3895	−0.0503	49.8940	0.9914
0.47	1.9507	0.5385	4115.2344	−542.3736	−0.0463	44.8805	0.9918
0.55	1.8902	0.5794	4173.0389	−587.5164	−0.0410	38.4204	0.9904
0.62	1.7329	0.6044	4341.0801	−615.4615	−0.0262	20.8704	0.9946
0.69	1.6694	0.6180	4401.4446	−630.7493	−0.0140	6.3494	0.9940

**Fig. 6:**
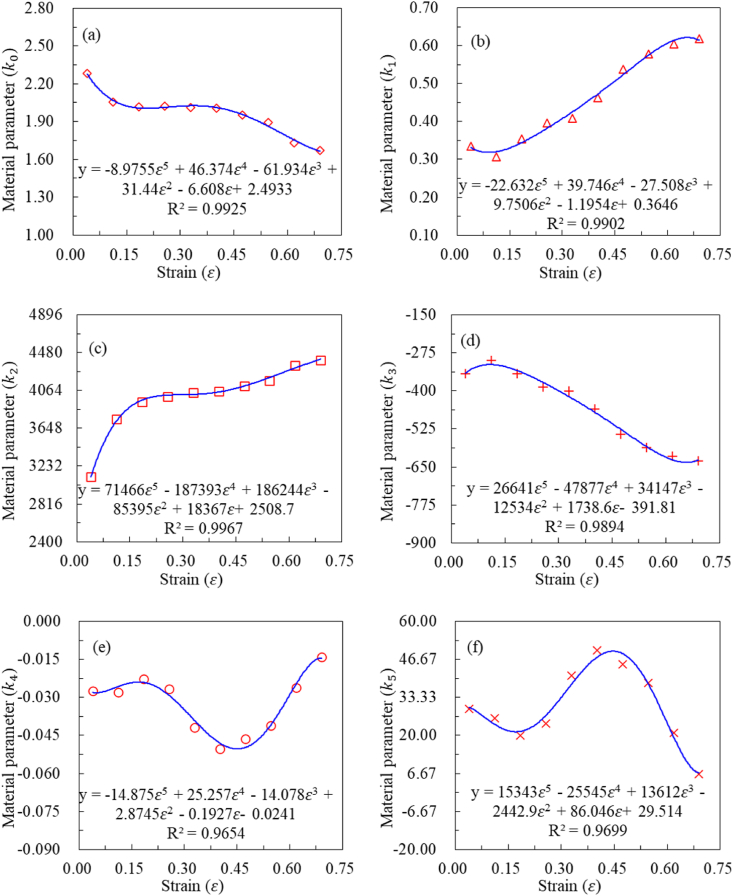
All the material parameters and its fitted curve: (a) $k_{0}-\varepsilon$, (b) $k_{1}-\varepsilon$, (c) $k_{2}-\varepsilon$, (d) $k_{3}-\varepsilon$, (e) $k_{4}-\varepsilon$, (f) $k_{5}-\varepsilon$.

As shown in Table 4, the coefficient of determination (R-squared) for all material parameters exceeds 0.9860 across various strain levels. This indicates that Eq. (5) exhibits a high level of regression accuracy for the experimental data. This high R-squared value confirms that our model effectively captures the relationship between stress, strain rate, and temperature, providing a reliable representation of the material's behavior. This accuracy is crucial for predicting performance and optimizing processing conditions in practical applications. Based on these regression results, the explicit expression for constitutive equation can be established. The final step to establish the explicit equation involves using a polynomial to fit each material parameter with strain at all strain levels. Table 5 presents the coefficients of the fitted fifth-degree polynomial, and the corresponding fitted plots are illustrated in Fig. 6 (a)-**(f)**.

**Table 5:** Material parameter polynomial coefficients.

Parameters	$\varepsilon^{5}$	$\varepsilon^{4}$	$\varepsilon^{3}$	$\varepsilon^{2}$	ε	Constant
$k_{0}$	−8.975	46.3744	−61.934	31.439	−6.608	2.493
$k_{1}$	−22.631	39.7458	−27.508	9.750	−1.195	0.364
$k_{2}$	71465.712	−187393.316	186244.289	−85395.37	18367.008	2508.708
$k_{3}$	26641.075	−47877.083	34147.203	−12533.64	1738.647	−391.805
$k_{4}$	−14.874	25.257	−14.077	2.874	−0.192	−0.024
$k_{5}$	15342.948	−25545.497	13612.262	−2442.907	86.046	29.514

As presented in Fig. 6(a)–(f), the R-squared for all material parameters exceeds 0.9654. This suggests that the fifth-degree polynomial effectively captures the dependence of material parameters on strain. The formula presented in Fig. 6(a)–(f) can be integrated into Eq. (5) to derive the explicit constitutive model.

#### 3.1.2 Classical model

The HS model has gained extensive application within simulation software, such as Forge NxT, for hot forming due to its simple formulation (explicit equation) and the ease of obtaining material parameters through multiple linear regression [[16], [17], [18]]. The general expression of the HS model is as shown in Eq. (6).

(6) $$\sigma =\text{Aexp}\left(w_{1}T\right)\varepsilon^{w_{2}}{\dot{\varepsilon }}^{w_{3}}\;\exp\left(\frac{w_{4}}{\varepsilon }\right){\left(1+\varepsilon \right)}^{w_{5}T}\;\exp\left(w_{6}\varepsilon \right){\dot{\varepsilon }}^{w_{7}T}T^{w_{8}}$$

where ε, $\dot{\varepsilon }$, σ, T is the strain, strain rate, stress, and temperature, respectively. A and $w_{1}-w_{8}$ are materials parameters. Eq. (7) is obtained by taking the natural logarithm of both sides of Eq. (6).

(7) $$\ln\;\sigma =\ln\;A+w_{1}T+w_{2}\;\ln\;\varepsilon +w_{3}\;\ln\dot{\varepsilon }+\frac{w_{4}}{\varepsilon }+w_{5}T\;\ln\left(1+\varepsilon \right)+w_{6}\varepsilon +w_{7}T\;\ln\dot{\varepsilon }+w_{8}\;\ln\;T$$

A linear relationship exists among serval variables, including logarithmic stress (lnσ), temperature (T), logarithmic strain (lnε), logarithmic strain rate ($\ln\dot{\varepsilon }$), reciprocal of strain (1/ε), product of temperature and logarithmic 1+ε (Tln(1+ε)), strain (ε), the product of temperature and logarithmic strain rate ($T\;\ln\dot{\varepsilon }$), and logarithmic temperature (lnT). The material constants in Eq. (7) can be identified by implementing multiple linear regression with the data provided in Table 2. In Table 2, the strain has been divided into 10 intervals, ranging from 0.04 to 0.69. By applying the discrete data from Table 2 to Eq. (7) and utilizing the least squares method for multiple linear regression, the material parameters are systematically identified, as presented in Table 6.

**Table 6:** Results of multiple linear regression for material parameters.

$w_{1}$	$w_{2}$	$w_{3}$	$w_{4}$	$w_{5}$	$w_{6}$	$w_{7}$	$w_{8}$	lnA
−0.00599	0.396069	−0.31338	−0.00064	−0.0015	0.192249	0.000336	4.139898	−15.9184

#### 3.1.3 Accuracy analysis

**Fig. 7:**
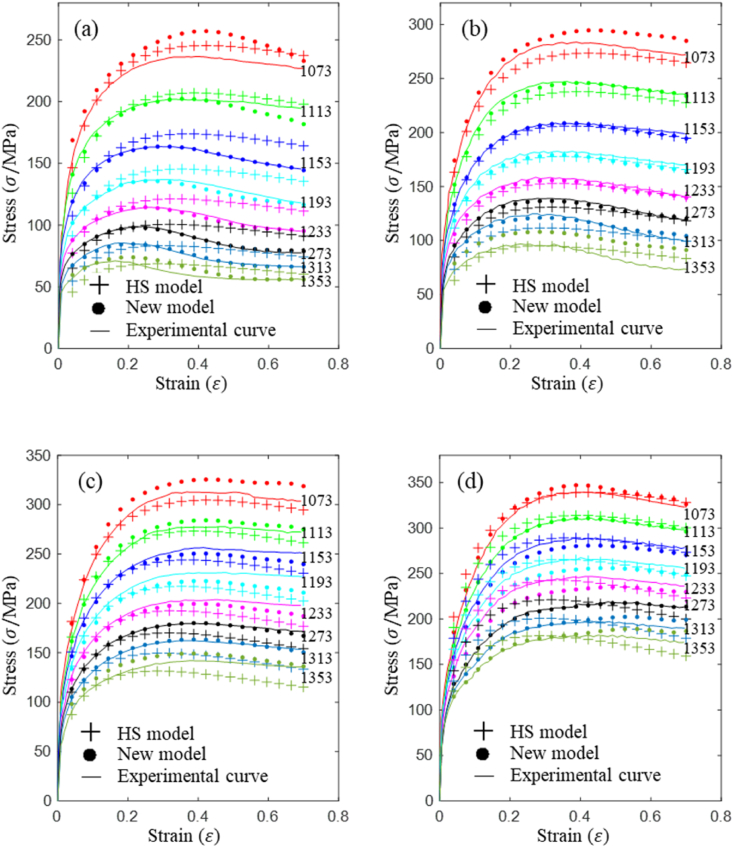
Comparison between experimental curves and predicted data from HS and new model: (a) 0.01 s^−1^, (b) 0.1 s^−1^, (c) 1 s^−1^, (d) 10 s^−1^.

Fig. 7 shows a comparison of the predicted data from both the new model and the HS model with the experimental data to assess prediction accuracy. In Fig. 7, solid lines are experimental data, ‘+’ symbols denote the predicted values from the HS model, and ‘•’ symbols indicate the predicted values from the new model.

Fig. 7(a)–(d) indicates that the new model demonstrates significantly higher overall prediction accuracy compared to the HS model. Fig. 7(a) shows that, at a strain rate of 0.01 s⁻^1^, the HS model's predicted values do not exhibit a significant softening effect during the softening stage, which contrasts with the experimental data. This indicates that the HS model has limited predictive accuracy for the softening effect of A100 steel at low strain rates. Fig. 7(b)–(d) show that the predicted values from the HS model during the softening stage are consistently lower than the experimental data, indicating that the HS model underestimates the softening effect in the high strain rate region. Because the HS model is based on a limited set of 8 material constants, it has fewer degrees of freedom, which restricts its predictive capabilities for A100 steel across varying strain rates, strains, and temperatures.

The prediction accuracy of the new model exceeds that of the HS model, with only a slight decrease in accuracy under specific conditions, such as a strain rate of 0.01 s⁻^1^ at 1073 K, and a strain rate of 1 s⁻^1^ at 1353 K.

**Fig. 8:**
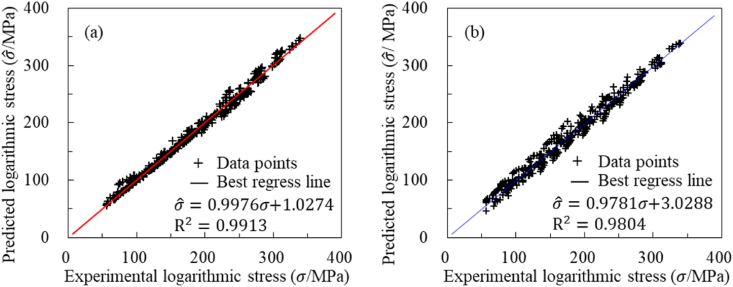
Correlation comparison between experimental and predicted stress: (a) New model, (b) HS model.

Fig. 8 (a) and (b) reveal three important findings. The first finding is related to the multiple coefficients of determination (R^2^), which is 0.9913 for the new model and 0.9804 for the HS model. The second finding pertains to the slope of the best regression line, where the new model has a slope of 0.9976, compared to 0.9781 for the HS model. Additionally, the intercepts are 1.0274 for the new model and 3.20288 for the HS model. The third finding is that the new model exhibits a significantly lower degree of dispersion compared to the HS model. Together, these observations confirm that the predictive accuracy of the new model is significantly higher than that of the HS model.

### 3.2 Hot processing maps and microstructure

#### 3.2.1 Hot processing maps

The hot processing maps of A100 steel can be established based on the new constitutive equation. According to the dynamic materials model established by Prasad et al. [19,20], the hot deformation process in a metal material involves power dissipation, where the total energy applied to workpiece (P) is partitioned into two distinct components: 1) power utilized for plastic deformation (G); and 2) power consumed for microstructural evolution (J). Eq. (8) provides the mathematical description of the above relationship.

(8) P=G+J

where $P=\sigma \dot{\varepsilon }$, *G=* $\int_{0}^{\dot{\varepsilon }}\sigma d\dot{\varepsilon }$ and $J=\int_{0}^{\sigma }\dot{\varepsilon }d\sigma$. Strain rate sensitivity is the ratio of dG to dJ, and can be computed using Eq. (9).

(9) $$m=\frac{dJ}{dG}=\frac{\dot{\varepsilon }d\sigma }{\sigma d\dot{\varepsilon }}=\frac{\text{dln}\sigma }{\text{dln}\dot{\varepsilon }}$$

The energy dissipation rate (*η*) plays a crucial role in quantifying the energy allocated to microstructural evolution within the realm of nonlinear energy dissipation [21]. A higher energy dissipation rate corresponds to a more substantial energy expenditure on microstructure evolution, consequently leading to more pronounced changes in microstructure morphology. Eq. (10) can be used to calculate the energy dissipation rate.

(10) $$\eta =2\left[1-\frac{1}{\sigma \dot{\varepsilon }}\left({\frac{\sigma \dot{\varepsilon }}{1+m}|}_{\dot{\varepsilon }={\dot{\varepsilon }}_{\min}}+\int_{{\dot{\varepsilon }}_{\min}}^{\dot{\varepsilon }}\sigma d\dot{\varepsilon }\right)\right]$$

According to Narayan's study [22,23], unstable deformation occurs in the system when the rate of entropy generation does not align with the rate of entropy consumption. When the material is undergoing unstable deformation, the over burning, shear bands, cracks and other phenomena will occur. The criterion is given by Eq. (11).

**Fig. 9:**
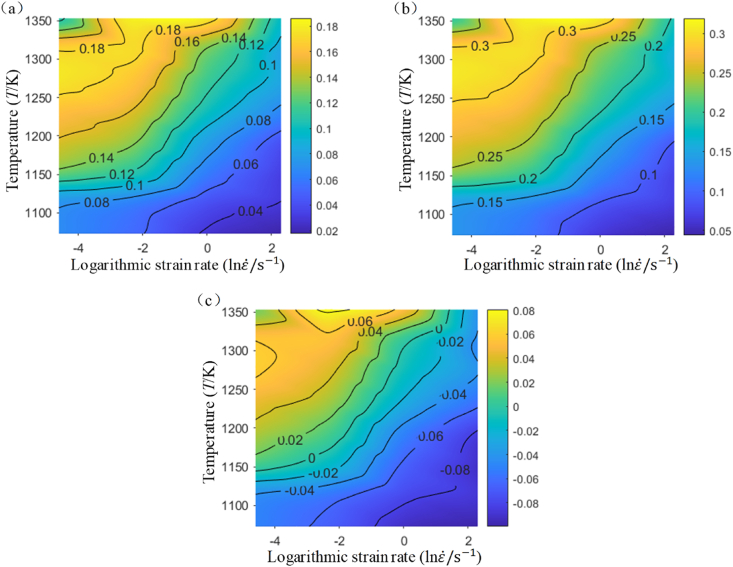
Distribution of strain rate sensitivity m (a), energy dissipation η (b), and instability factor ξ (c) at a strain of 0.6900.

(11) ξ=2m−η<0

where ξ is the instability factor. Next, the analytical formulas for the energy dissipation rate and the instability factor can be derived by substituting Eq. (5) into Eq. (9), **(10)**, and **(11)**. The hot processing maps corresponding to a strain level of 0.69 have been illustrated in Fig. 9.

Fig. 9 (a), **(b)**, and **(c)** show that, at the same strain level, the distribution patterns of strain rate sensitivity, energy dissipation rate, and instability factor with respect to temperature and logarithmic strain rate are similarly patterned. Furthermore, as strain increases, temperature rises, and strain rate decreases, the values of strain rate sensitivity, energy dissipation rate, and instability factor all show an increasing trend.

#### 3.2.2 Microstructure

Microstructure analysis was conducted on the central region of each sample after hot compression and water quenching to investigate the evolution of the microstructure of A100 steel under various hot compression conditions and to validate the accuracy of the hot processing maps. The resulting microstructure diagrams as shown in Table 7.

**Table 7:**
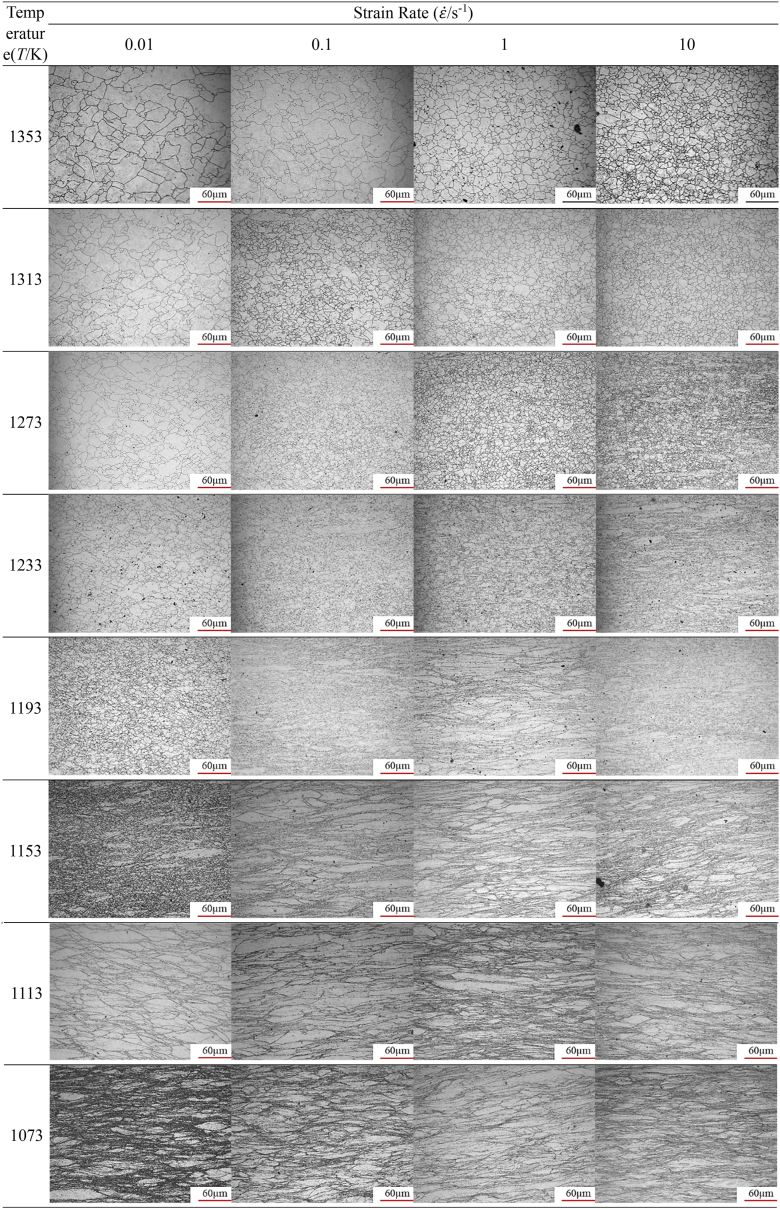
The microstructures under different experimental conditions at strain 0.69.

When the temperature is below 1233K and the strain rate exceeds 0.1s^−1^, there are many original coarse grains. Additionally, as temperature decreases and strain rate increases, the number of original coarse grain structures also increases. These rules align with the conclusions drawn from the hot processing diagram: specifically, lower temperatures and higher strain rates are associated with reduced energy dissipation rates, leading to lower energy consumption during the material's microstructural evolution. Consequently, in these scenarios, most of the energy is expended in the plastic deformation of microscale grains. These findings further validate the hot processing maps and underscore the significant influence of temperature and strain rate on the material's microstructural evolution.

As shown in Table 7, when strain and strain rate are constant, the average grain size decreases initially and then increase with increasing temperature. The average gain size for initial specimen is about 50 μm (Fig. 2). This is attributed to the evolution process of the microstructure of hot-compressed samples with temperature increase, which goes through the stages of incomplete dynamic recrystallization (retaining original coarse grain structures) → complete dynamic recrystallization → grain growth. In addition, there are three rules for A100 steel from Table 7, Table 8. Firstly, when the compression experiment is conducted at low temperatures (T≤1193K), the average grain size decreases with decreasing strain rate. Secondly, when the compression experiment is conducted at high temperatures (T≥1313K), the average grain size increases with decreasing strain rate. Finally, when the compression experiment is medium temperature (1233K≤T≤1273K), the average grain size decreases initially and then increase with decreasing strain rate. The reason for this phenomenon is that at low temperatures, the energy of metal atoms (thermal energy) is low, the nucleation rate is low, and the lower strain rate can prolong the recrystallization time during the deformation process, thereby increasing the number of recrystallizations, and thus reducing the average grain size. At medium temperature, the energy of metal atoms (thermal energy) is higher, the nucleation rate is larger, and nucleation and recrystallization can be completed at higher strain rate. Therefore, at low strain rate, the grain size will grow, and then the average grain size will first decrease and then increase with the decrease of strain rate. At high temperature, the energy of metal atoms (thermal energy) is extremely high, and the nucleation rate is extremely high. Even at high strain rate, the nucleation and recrystallization growth process can be completed. As the strain rate decreases, the degree of grain growth increases, and the average grain size gradually becomes larger.

**Table 8:**
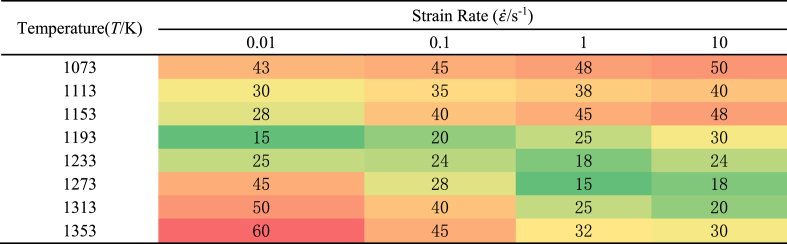
Average gain size under different experimental conditions at strain 0.69.

**Fig. 10:**
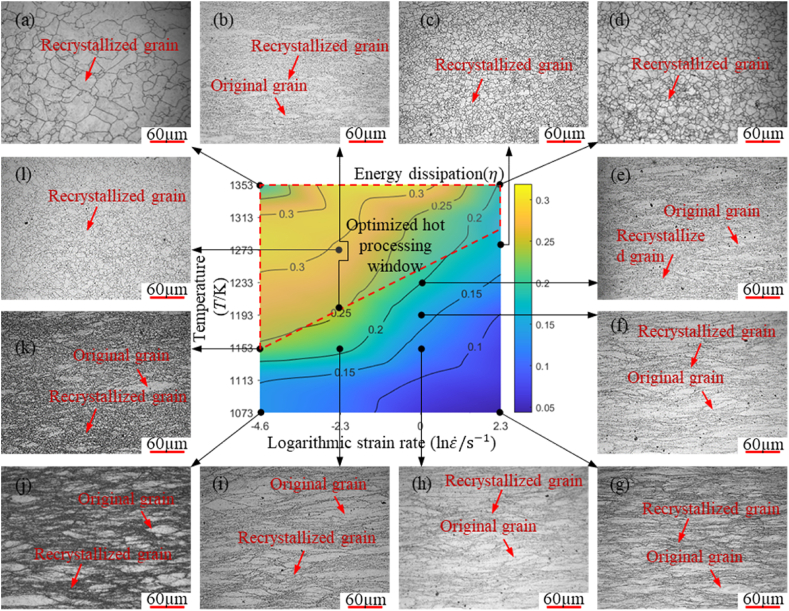
Microstructures corresponding to typical regions on the energy dissipation diagram.

To visually analyze the relationship between microstructure and energy dissipation rate, the microstructures corresponding to typical hot compression conditions were plotted on an energy dissipation diagram, yielding Fig. 10.

As shown in Fig. 10(a)–(g), within the experimental temperature and strain rate range for hot deformation of A100 steel, the metal exhibits stable deformation without phenomena such as adiabatic shear bands or localized deformations. As shown in Fig. 10 (e)–(k), in regions of unreasonable hot deformation, the microstructure of the metal appears fibrous, which is an undesirable material state in hot deformation. Microstructure analysis reveals that when the energy dissipation rate exceeds 25 %, A100 steel can undergo dynamic recrystallization effectively, resulting in a uniform equiaxed grain structure after deformation. The optimized hot processing window for A100 steel is outlined by the dashed lines in Fig. 10, covering temperatures from 1153 to 1353K and strain rates from 0.01 to 10 s^−1^.

## 4 Conclusions

The hot deformation behaviors and the hot processing map for A100 steel are researched based on flow stress measured from hot compression experiments conducted at temperatures spanning 1073–1353 K and strain rates ranging from 0.01 to 10 s^−1^. Microstructure analyses were conducted to validate the hot processing map of A100 steel. Three key conclusions are listed as follows:

(1) The factors influencing stress are ranked in the following order of significance: strain rate, temperature, strain, the interaction between strain and strain rate, the interaction between strain and temperature, and the interaction between strain rate and temperature. All factors have a significant impact on stress, with strain rate, temperature, and strain being the most influential.
(2) A strong linear relationship exists between the logarithmic stress and the reciprocal of temperature, while a robust quadratic relationship is observed between logarithmic stress and logarithmic strain rate. The new constitutive model for A100 steel is expressed as: $\ln\;\sigma =k_{0}+k_{1}\;\ln\dot{\varepsilon }+k_{2}\frac{1}{T}+k_{3}\frac{1}{T}\ln\dot{\varepsilon }+k_{4}\;\ln^{2}\dot{\varepsilon }+k_{5}\frac{1}{T}\ln^{2}\dot{\varepsilon }$. This model incorporates both linear and quadratic terms to accurately capture the complex interactions between stress, strain rate, and temperature. The inclusion of these terms allows for a nuanced understanding of how different factors interplay, providing a robust framework for predicting material behavior under various conditions. The new constitutive model, with a correlation coefficient of 0.9913, shows exceptional predictive capability for the flow behavior of A100 steel.
(3) Microstructural analysis reveals that effective DRX occurs when energy dissipation rate exceeds 25 %, leading to a uniform equiaxed grain structure after deformation. This finding is significant as it highlights the critical threshold for effective recrystallization, which is essential for optimizing material properties. Understanding this relationship enables the control of A100 steel's microstructural evolution during hot processing, thereby achieving the desired mechanical properties. Unstable regions predominantly occur in zones with high strain rates and low temperatures, where the development of coarse, elongated microstructures adversely impacts the mechanical properties. It is crucial to avoid these conditions during the hot deformation of A100 steel. The optimal hot processing windows for A100 steel fall within a temperature range of 1153–1353K and a strain rate range of 0.01–10 s^−1^, where complete DRX is achieved.

## CRediT authorship contribution statement

**Chaoyuan Sun:** Writing – original draft, Conceptualization. **Yi Qin:** Investigation. **Yang Liu:** Data curation. **Guiqian Xiao:** Software, Data curation. **Jie Zhou:** Supervision. **Jiansheng Zhang:** Investigation, Funding acquisition, Conceptualization.

## Data availability statement

No additional data was used for the research described in the article.

## Funding statement

This research is supported by the 10.13039/501100001809National Natural Science Foundation of China (No. 52205337), General Project of Chongqing Natural Science Foundation (No. CSTB2024NSCQ-MSX0702), the Chongqing Special Project for Technological Innovation and Application Development (No. cstc2022ycjhbgzxm0224).

## Declaration of competing interest

There is no competing interest.
